# Golgi α1,2-mannosidase I induces clustering and compartmentalization of CD147 during epithelial cell migration

**DOI:** 10.1080/19336918.2020.1764170

**Published:** 2020-05-18

**Authors:** Miguel Gonzalez-Andrades, Supriya S. Jalimarada, Maria Rodriguez-Benavente, Marissa N. Feeley, Ashley M. Woodward, Dina B. AbuSamra, Pablo Argüeso

**Affiliations:** Schepens Eye Research Institute of Massachusetts Eye and Ear, Department of Ophthalmology, Harvard Medical School, Boston, MA, USA

**Keywords:** Cell migration, CD147, epithelia, α1,2-mannosidase I, N-glycosylation

## Abstract

CD147 is a widely expressed matrix metalloproteinase inducer involved in the regulation of cell migration. The high glycosylation and ability to undergo oligomerization have been linked to CD147 function, yet there is limited understanding on the molecular mechanisms behind these processes. The current study demonstrates that the expression of Golgi α1,2-mannosidase I is key to maintaining the cell surface organization of CD147 during cell migration. Using an in vitro model of stratified human corneal epithelial wound healing, we show that CD147 is clustered within lateral plasma membranes at the leading edge of adjacent migrating cells. This localization correlates with a surge in matrix metalloproteinase activity and an increase in the expression of α1,2-mannosidase subtype IC (MAN1C1). Global inhibition of α1,2-mannosidase I activity with deoxymannojirimycin markedly attenuates the glycosylation of CD147 and disrupts its surface distribution at the leading edge, concomitantly reducing the expression of matrix metalloproteinase-9. Likewise, treatment with deoxymannojirimycin or siRNA-mediated knockdown of MAN1C1 impairs the ability of the carbohydrate-binding protein galectin-3 to stimulate CD147 clustering in unwounded cells. We conclude that the mannose-trimming activity of α1,2-mannosidase I coordinates the clustering and compartmentalization of CD147 that follows an epithelial injury.

## Introduction

CD147 is a glycosylated type I transmembrane protein known to induce cell migration in several physiological and pathological processes such as development, tissue repair, or cancer [1,2]. It was first described in 1982 as an inducer of matrix metalloproteinases (MMPs), a family of extracellular endopeptidases long thought to be involved in cell migration by breaking down matrix barriers and plasma membrane proteins [3,4]. Since then, additional evidence has demonstrated that homo-oligomerization of CD147 is necessary to prompt MMP production, both through homotypic and heterotypic cell interactions [5]. An important characteristic of mature CD147 is that almost half its size is made up of carbohydrates as a result of heterogeneous N-glycosylation [6]. Only the most highly glycosylated forms of CD147 function to induce cell surface clustering and MMP production [7] but the precise molecular details have yet to be determined.

The glycosylation of CD147 starts in the endoplasmic reticulum with the transfer of a dolichol phosphate oligosaccharide to conserved asparagine residues in the nascent polypeptide, resulting in an immature high-mannose precursor [8]. Glucosidases, mannosidases, and glycosyltransferases subsequently modify this precursor in the lumen of the endoplasmic reticulum and Golgi to generate different types of N-glycans based on the content of mannose and the presence of branching antennae. The Golgi α1,2-mannosidase I family of enzymes (MAN1A1, MAN1A2, and MAN1C1) are critical in this process as they cleave high-mannose structures to allow glycan maturation [9]. Mass spectrometry analysis of purified native CD147 from human lung cancer tissue has demonstrated the presence of both high-mannose type and complex-type N-linked oligosaccharides [10]. Among the three N-glycosylation sites present on the extracellular region of CD147, Asn152 appears to play an important role in ensuring proper protein folding. Mutation of this amino acid results in retention of CD147 in the endoplasmic reticulum and the activation of the unfolded protein response, with subsequent impairment of its ability to induce MMP synthesis [10,11].

The degree of glycosylation of transmembrane proteins is critical to regulate surface interactions following translocation into the plasma membrane and, consequently, their biological activities [12]. A number of binding partners have been identified for CD147 within the same cell or in adjacent cells that include monocarboxylate transporters and platelet glycoprotein VI, respectively [13]. It is the glycosylated portion of CD147 that serves as a ligand for lectins such as galectin-3 and E-selectin. Galectin-3 is a β-galactoside-binding protein that promotes the clustering and co-localization of CD147 and integrin-β1 on retinal pigment epithelial cells [14]. In cornea, galectin-3 induces MMP9 expression and regulates epithelial rearrangements at points of cell–cell contact by clustering CD147 on the cell surface [15]. It has been hypothesized that structural variations in the glycosylation of CD147 could alter its interaction with carbohydrate-binding proteins and, in turn, modulate the activities of CD147.

N-glycosylation is essential for the biological events that mediate cell adhesion and migration in corneal epithelium. More than 30 y ago, Gipson et al. demonstrated the requirement of N-glycosylation for the continued migration of epithelial sheets during corneal wound healing [16]. Glycogene microarrays have subsequently identified a number of glycosyltransferases and glycan degradation enzymes differentially expressed in response to corneal injury [17]. However, the precise role of these enzymes in modulating the activities of glycoproteins relevant to epithelial cell motility has not been completely delineated. In this work, we use an *in vitro* model of wound healing to show that CD147 is clustered within lateral plasma membranes of migrating cells within areas of high gelatinolytic activity. We find that Golgi α1,2-mannosidase I is critical for maintaining the cell surface organization of CD147 during cell migration and demonstrate that the abrogation of MAN1C1 impairs the ability of exogenous galectin-3 to prompt CD147 clustering. We conclude that the mannose-trimming activity of α1,2-mannosidase I coordinates the clustering and compartmentalization of CD147 during epithelial cell migration.

## Results

### CD147 is restricted to the lateral plasma membrane in migrating human corneal epithelial cells

**Figure 1.**
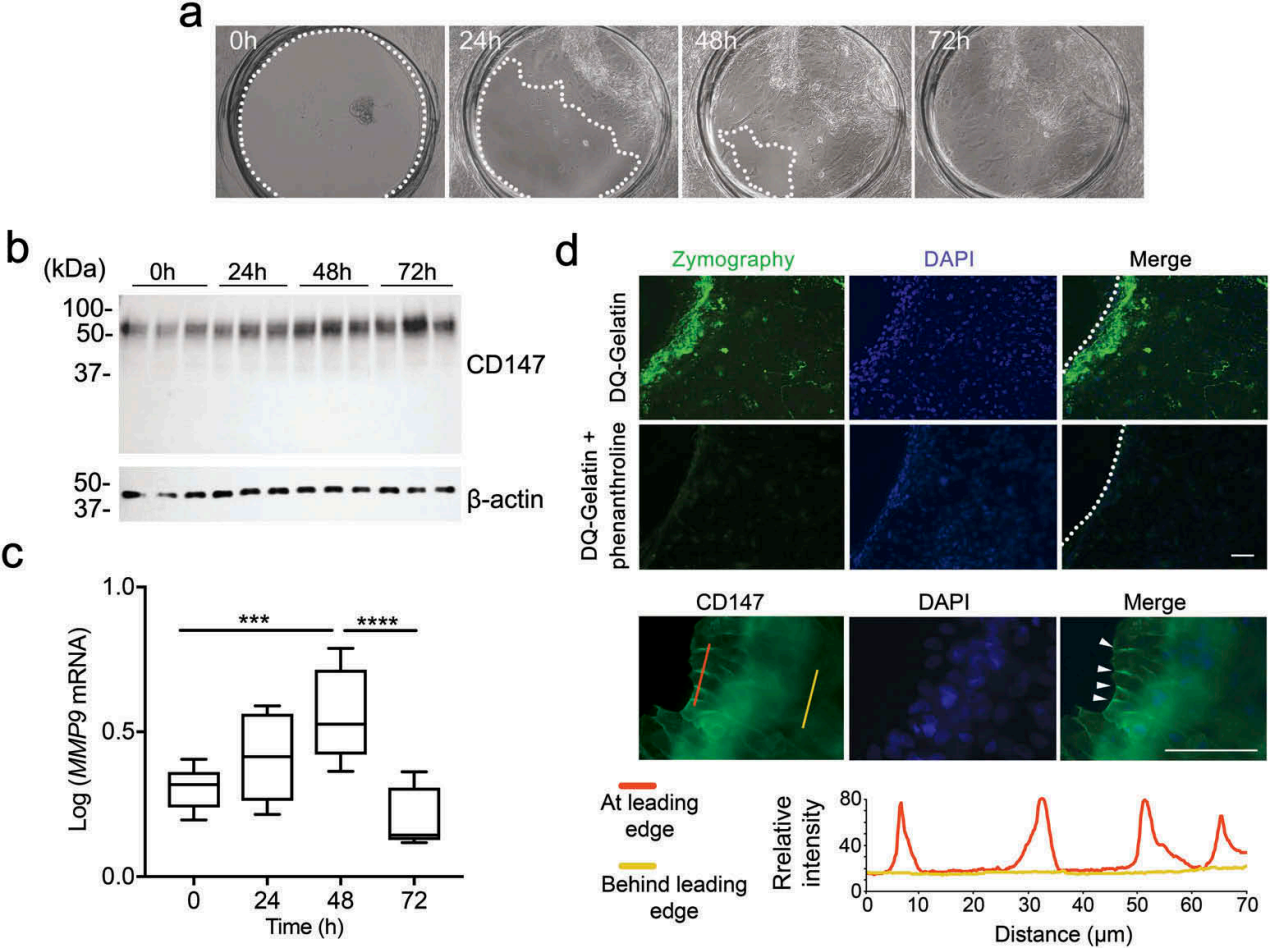
CD147 localizes to lateral plasma membranes in migrating corneal epithelial cells. (a) Circular wounds of 1.0 mm in diameter were made on stratified cultures of human corneal epithelial cells. Re-epithelialization was monitored for 72 h. (b) Multiple wounds were created on individual wells using a 33-hole punch template. The presence of CD147 in epithelial cell lysates collected at different time points was assessed by immunoblot. (c) Same as (b) except that total RNA was extracted to evaluate *MMP9* expression by qPCR. Experiments were performed at least in triplicate. (d) Multiple wounds were created on glass coverslips using an 8-hole punch template and analyzed 24 h later. The gelatinolytic activity was assessed by in situ zymography. The cellular distribution of CD147 was determined by immunofluorescence. Clustering within lateral plasma membranes at the leading edge is shown with arrowheads. Line-intensity scan analyses for CD147 in epithelial cells located at, and behind, the leading edge were performed using ImageJ software. Nuclei were visualized with DAPI. The box and whisker plots show the 25 and 75 percentiles (box) and the median and the minimum and maximum data values (whiskers). Significance was determined using one-way analysis of variance with Tukey’s post hoc test. ***p < 0.001; ****p < 0.0001. Scale bars, 100 μm.

A significant characteristic of CD147 is its high level of glycosylation, which accounts for approximately 50% of its mass. In this study, we sought to identify the glycogenes that regulate CD147 glycosylation in human corneal epithelial cells and their contribution to CD147 clustering and compartmentalization following epithelial injury. For this purpose, we employed an established *in vitro* model of stratified human epithelial wound healing [18]. Direct visualization of the migratory process in this model by time-lapse microscopy revealed a considerable migration of epithelial cells into the wounded area at 48 h and complete re-epithelialization at 72 h (Figure 1a). Immunoblotting experiments demonstrated that closure of the wound was associated with an increase in the highly glycosylated form of CD147, as evidenced by the presence of a band at approximately 60 kDa (Figure 1b). This increase was concomitant with an upregulation in the number of *MMP9* transcripts and the accumulation of gelatinolytic activity within the leading edge of the migrating epithelium (Figure 1c,d). Importantly, immunostaining revealed clustering of CD147 within cell-cell junctions at the leading edge of adjacent migrating cells (Figure 1d), consistent with its function as a modulator of migration activity in epithelia.

### *MAN1C1* expression increases after epithelial wounding

**Figure 2.**
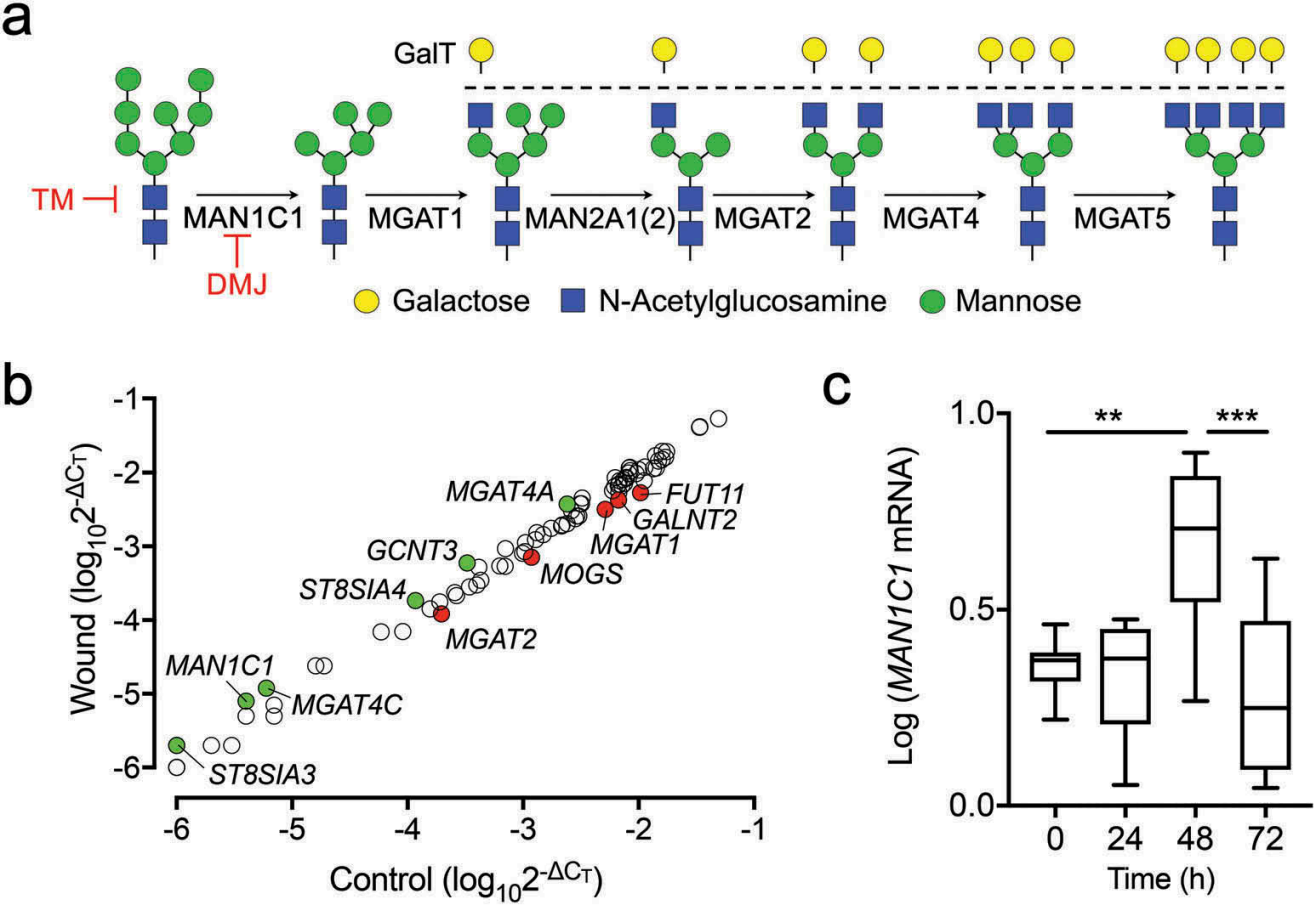
*MAN1C1* expression increases after epithelial wounding. (a) Schematic diagram of N-glycan branches and enzymes responsible for their synthesis in the Golgi compartment. TM, tunicamycin; DMJ, deoxymannojirimycin. (b) Multiple wounds were created on individual wells using a 33-hole punch template. Total RNA was extracted 48 h later to examine the transcriptional levels of known N-glycosylation enzymes in stratified human corneal epithelial cells using a pathway-focused PCR array. The green and red dots indicate at least 1.5-fold up- or down-regulation, respectively, compared to unwounded control. Experiments were performed in duplicate. (c) Same as (b) except that total RNA was extracted from the leading edge using a 2.0 mm punch concentric to the wound (n = 20 wounds/sample). The presence of *MAN1C1* at different time points was assessed by qPCR. Experiments were performed at least in triplicate. The box and whisker plots show the 25 and 75 percentiles (box) and the median and the minimum and maximum data values (whiskers). Significance was determined using one-way analysis of variance with Tukey’s post hoc test. **p < 0.01; ***p < 0.001.

The branching and maturation of N-glycans require the removal of terminal mannose and the addition of N-acetylglucosamine and galactose during passage through the Golgi (Figure 2a). Because of the important role of this pathway in CD147 function, we performed a pathway-focused PCR analysis to investigate the transcriptional levels of known human glycosylation enzymes in migrating corneal epithelial cells. A direct comparison of 84 genes in the array demonstrated that the relative expression of 11 glycosyltransferase and glycosidase enzymes was altered by more than 1.5-fold in wounded cell cultures compared to control (Figure 2b). Here, it is important to note that multiple wounds were performed in the cell culture dish to increase the ratio of damaged to undamaged cells. The expression of *MAN1C1, MGAT4A,* and *MGAT4 C*, which encode Golgi enzymes involved in the trimming of mannose and the production of tri- and tetra-antennary N-glycans was upregulated, whereas *MGAT1* and *MGAT2* involved in the production of mono- and bi-antennary N-glycans were downregulated. Other enzymes that were found to be differentially expressed included sialyltransferases (*ST8SIA3, ST8SIA4*), mucin-type O-glycosyltransferases (*GCNT3, GALNT2*), a fucosyltransferase (*FUT11*) and mannosyl-oligosaccharide glucosidase (*MOGS*). We focused our attention on MAN1C1 since Golgi α1,2-mannosidase I activity is necessary for initiating the maturation of N-glycans. Barely expressed in unwounded conditions (Supplementary Table S1), expression analysis by qPCR demonstrated that the number of *MAN1C1* transcripts increased 48 h post wounding and returned to baseline at 72 h concomitantly with the closure of the wounded area (Figure 2c).

### Inhibition of Golgi α1,2-mannosidase I impairs CD147 localization and function

**Figure 3.**
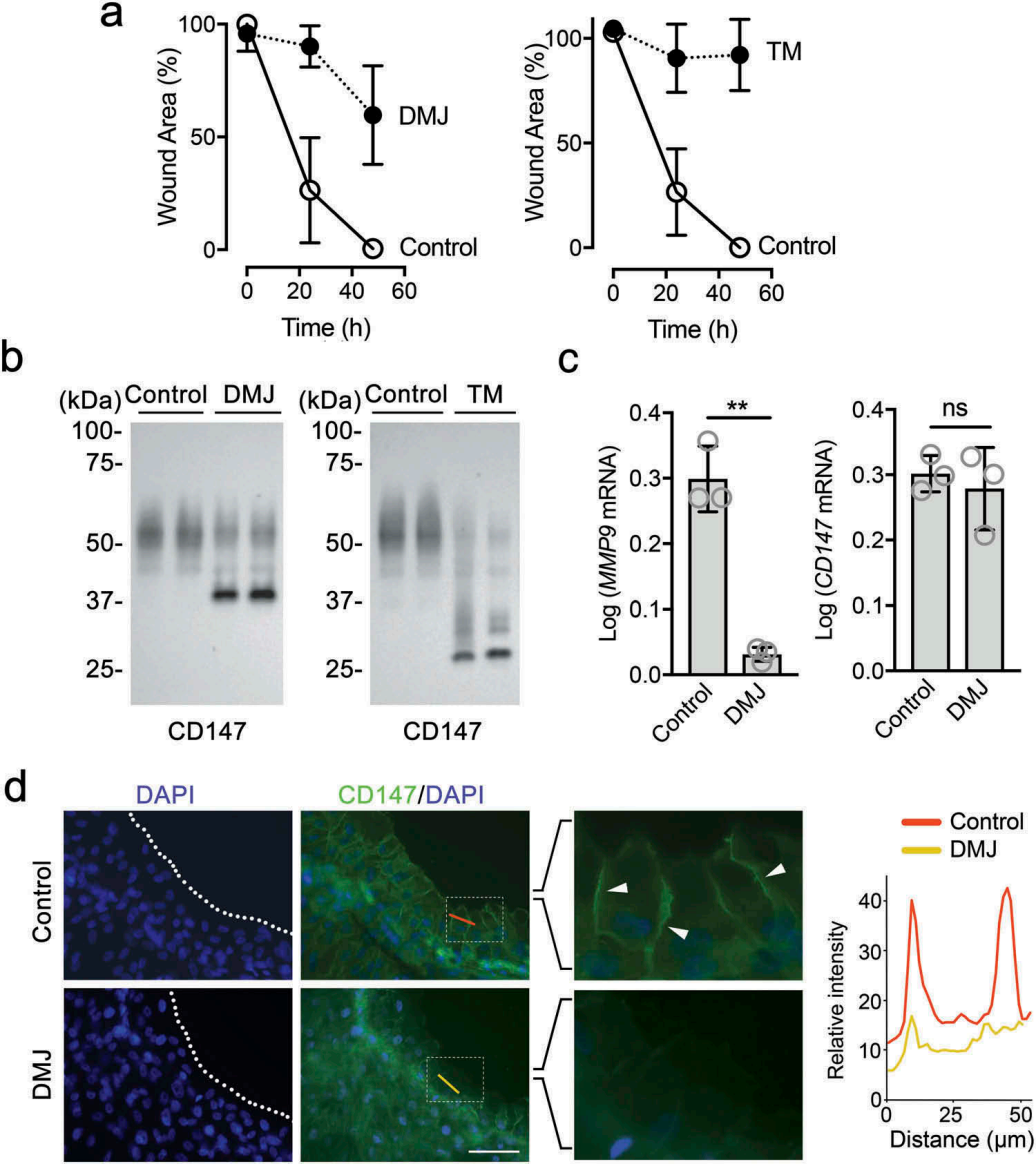
Inhibition of Golgi α1,2-mannosidase I impairs CD147 localization and function. (a) Morphometric analysis of the extent of re-epithelialization in the presence of culture media containing deoxymannojirimycin (DMJ), tunicamycin (TM), or no inhibitor (control). The number of wounds analyzed per time point was 23–48. (b) The electrophoretic migration of CD147 from stratified cultures of human corneal epithelial cells treated with glycosylation inhibitors was assessed by immunoblot. (c) Total RNA was extracted from the leading edge using a 2.0 mm punch concentric to the wound (n = 20 wounds/sample). The expression of *MMP9* and *CD147* was assessed by qPCR 48 h after wounding. Experiments were performed in triplicate. (d) The cellular distribution of CD147 was determined by immunofluorescence 24 h after wounding. Clustering within lateral plasma membranes at the leading edge is shown with arrowheads. Line-intensity scan analyses for CD147 in control cells and cells treated with DMJ were performed using ImageJ software. Nuclei were visualized with DAPI. Data are represented as the mean ± S.D. Significance was determined using the Student’s t-test. ns, not significant; **p < 0.01. Scale bar, 100 μm.

Next, we determined the effect of Golgi α1,2-mannosidase I inhibition on the cellular distribution of CD147 in migrating corneal epithelial cells. For this purpose, we used deoxymannojirimycin, a global α1,2-mannosidase I inhibitor that blocks the removal of mannose residues in the Golgi, leaving a high-mannose precursor that cannot be further processed [19]. We found that the rate of wound closure was significantly delayed following the addition of deoxymannojirimycin to the cell culture media (Figure 3a). This impairment was similar to that observed in cells cultured with tunicamycin, an antibiotic that blocks the first step in the formation of the dolichol phosphate precursor of N-glycans. We observed that deoxymannojirimycin produced a low glycosylated form of CD147, consistent with the loss of branched N-glycan structures, whereas tunicamycin effectively deglycosylated CD147, producing a protein band of approximately 30 kDa (Figure 3b).

The highly glycosylated species of CD147 are responsible for the induction of MMP biosynthesis through a process that requires homophilic interactions within the membrane or between cells [5,7]. MMP9 is expressed by migrating basal epithelial cells after wounding [20,21] and appears to have unique specific functions compared to other MMPs in cornea, such as control of cell replication [22]. The primary regulation of MMP9 activity is transcriptional and involves the activation of its promoter [23,24]. In our experiments, we found that inhibition of Golgi α1,2-mannosidase I with deoxymannojirimycin significantly reduced the number of *MMP9* transcripts in wounded cultures without affecting the level of *CD147* expression (Figure 3c), indicating that corneal epithelial cells require the processing of high-mannose precursors on CD147 in order to promote *MMP9* transcription. Furthermore, in these experiments, the ability of CD147 to cluster within lateral plasma membranes at the leading edge of adjacent migrating cells was impaired following treatment with deoxymannojirimycin (Figure 3d), suggesting that Golgi α1,2-mannosidase I activity is important in determining the correct localization of CD147 that allows *MMP9* expression.

### Golgi α1,2-mannosidase I regulates galectin-3-induced CD147 clustering

**Figure 4.**
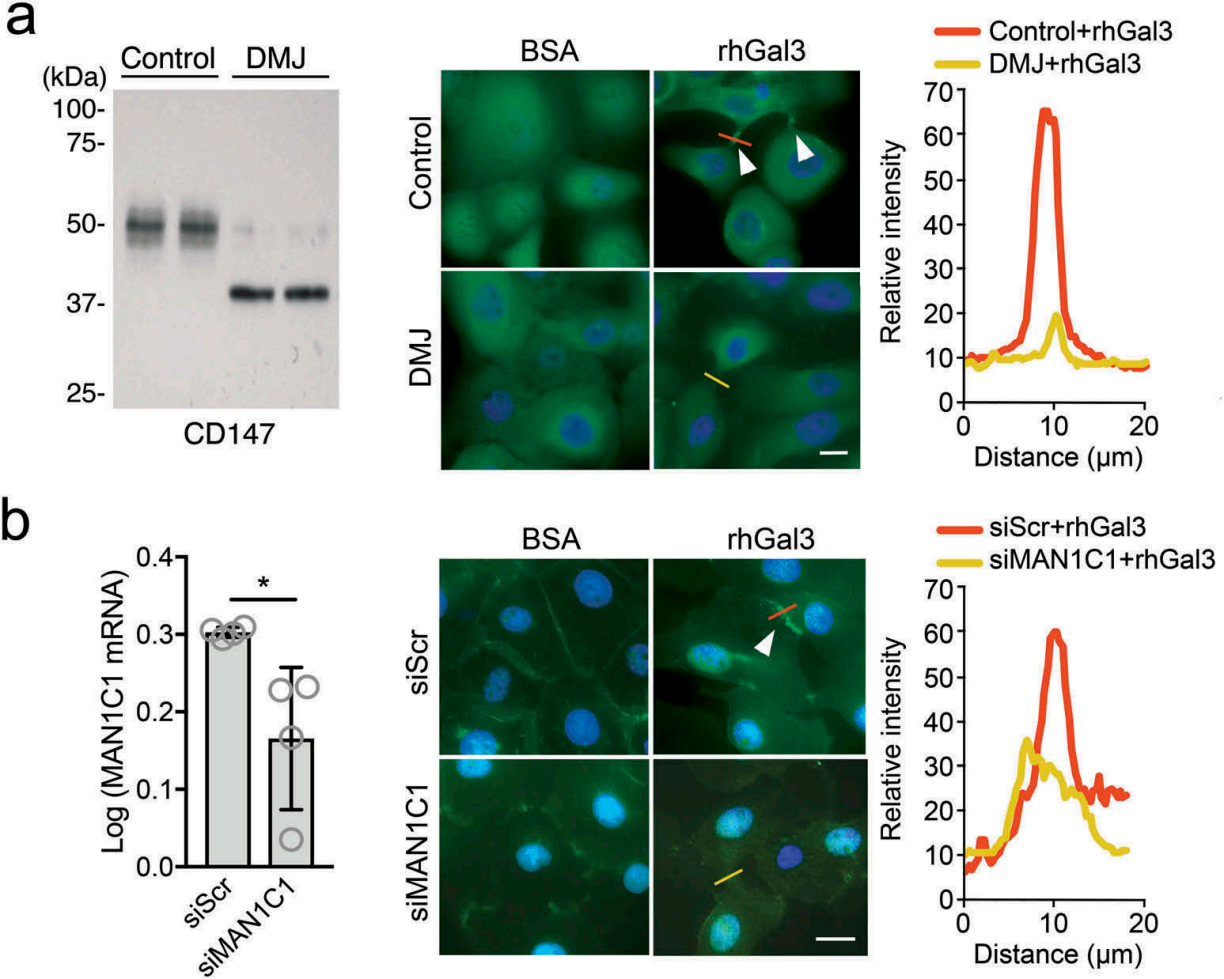
Golgi α1,2-mannosidase I regulates galectin-3-induced CD147 clustering. (a) The electrophoretic migration of CD147 from monolayer cultures of human corneal epithelial cells treated with deoxymannojirimycin (DMJ) was assessed by immunoblot. DMJ-treated cells were subsequently incubated with recombinant human galectin-3 (rhGal3) or bovine serum albumin (BSA) for 24 h. The cellular distribution of CD147 was determined by immunofluorescence. Enrichment of endogenous CD147 in clusters at cell–cell junctions is shown with arrowheads. Nuclei were visualized with DAPI. (b) Monolayer cell cultures were transfected with siRNA targeting *MAN1C1* (siMAN1C1) or negative control (siScr). Total RNA was extracted from the cultures 3 d later and the expression of *MAN1C1* was assessed by qPCR. Experiments were performed in triplicate. Following transfection, the cell cultures were treated with rhGal3 or BSA for 24 h and the cellular distribution of CD147 was determined by immunofluorescence. Line-intensity scan analyses for CD147 in (a) and (b) were performed using ImageJ software. Data are represented as the mean ± S.D. Significance was determined using Mann–Whitney test. *p < 0.05. Scale bars, 20 μm.

Galectin-3 is a carbohydrate-binding protein upregulated in migrating epithelia of healing corneas [25]. We have previously shown that galectin-3 in monolayer cultures of human corneal epithelial cells induces MMP9 expression in a manner that is dependent on the interaction with and clustering of CD147 on the cell surface [15]. Therefore, we investigated the role of Golgi α1,2-mannosidase I in mediating the clustering activities of galectin-3. To this purpose, we treated monolayer cultures of corneal epithelial cells with deoxymannojirimycin to produce a lowly glycosylated form of CD147 (Figure 4a). In agreement with previous results, we found that the addition of exogenous galectin-3 was sufficient to promote CD147 clustering within cell-cell junctions. On the other hand, galectin-3 failed to cluster CD147 in the presence of deoxymannojirimycin. In subsequent experiments, we used small interfering RNAs (siRNAs) to silence the expression of endogenous *MAN1C1* (Figure 4b). Once again, we found that the abrogation of *MAN1C1* but not a scramble control impaired the galectin-3-mediated clustering of CD147, supporting a role for Golgi α1,2-mannosidase I in facilitating the interaction between galectin-3 and CD147 on epithelial cell surfaces.

## Discussion

In response to injury, epithelial cells initiate a series of coordinated activities aimed to promote migration and restore the integrity of the affected tissue. The expression and clustering of CD147 on the cell surface are among those activities. By inducing MMPs, CD147 contributes to modify the extracellular environment and facilitate motility by breaking down components of the basement membrane [26,27]. In this report, we provide a mechanistic link between the mannose-trimming activity of Golgi α1,2-mannosidase I and the biological activities of CD147 during epithelial cell migration. We show that *MAN1C1* is overexpressed in an *in vitro* model of human corneal epithelial wound healing and that Golgi α1,2-mannosidase I contributes to promote *MMP9* expression and to maintain the cell surface organization of CD147. Moreover, we find that the suppression of *MAN1C1* negatively affects the ability of exogenous galectin-3 to prompt CD147 clustering in corneal epithelial cells.

Class I α1,2-mannosidases are a family of glycosidases with distinctive enzymatic properties depending on their subcellular localization. In the Golgi, these enzymes process Man_8-9_GlcNAc_2_ into Man_5_GlcNAc_2_, therefore providing the substrates required for the formation of branched N-glycans. There are three Golgi class I α1,2-mannosidases, MAN1A1, MAN1A2, and MAN1C1, which are expressed in a tissue- and cell-specific manner [28]. Despite a tremendous amount of work done in understanding the mannose-trimming activities of these enzymes, little is known about their significance to pathophysiological conditions. Initially reported in 2000 by Tremblay and Herscovics, MAN1C1 is expressed in a variety of human tissues such as liver, ovary, and intestine [29]. It has been suggested that MAN1C1 is a potential tumor suppressor [30,31]. Indeed, data obtained from two human renal cancer cell lines indicate that overexpression of MAN1C1 inhibits cell migration and decreases invasiveness [31]. This is in contrast with our results showing a positive correlation between cell migration and MAN1C1 expression in human corneal epithelial cells, which is further supported by our observation that inhibition of α1,2-mannosidase I activity with deoxymannojirimycin impairs cell migration. This apparent contradiction could potentially be explained by intrinsic differences between individual cell types and the diverse migratory patterns associated with wound healing and tumor progression [32]. Moreover, MAN1C1 might produce opposing responses in different tissues and pathological states as a result of variations in the mechanisms of glycan processing and the distinct availability of protein acceptors [33,34], possibilities that warrant further investigation.

It is becoming increasingly clear that glycan-modifying enzymes function to generate functional CD147 on plasma membranes. This is evidenced by MGAT5, a glycosyltransferase that catalyzes the addition of tetra-antennary N-glycan branches to CD147, resulting in a mature protein product [7]. Increased branching in hepatocarcinoma cells as a result of overexpression of MGAT5 causes enhanced trafficking of CD147 to the plasma membrane and the induction of MMP2 activity [10]. Similarly, overexpression of *MGAT4A* in hepatocarcinoma cells has been shown to promote CD147N-glycan branching and to enhance cell migration and metastatic capability [35]. These results are in line with our PCR array data showing that the expression of *MGAT4A* and *MGAT4C*, two genes encoding enzymes involved in the production of tri- and tetra-antennary N-glycans, is upregulated during corneal epithelial migration. The fact that *MAN1C1* also increases in our model further supports the concept that the processing of the immature high-mannose precursor is an essential regulatory step that precedes the formation of functional CD147. Interestingly, the presence of bi-antennary and bisecting N-glycan branches has been reported in CD147, although their biological significance remains unclear [10,35]. It has been suggested that reduced activity of the enzymes responsible for these structures is optimal for the addition of tetra-antennary N-glycan branches to glycoproteins [36,37]. This hypothesis would be consistent with the finding of reduced numbers of *MGAT1* and *MGAT2* transcripts in our *in vitro* model of corneal epithelial wound healing.

The presence of distinct structural motifs in CD147 allows binding to a number of proteins including cyclophilins, monocarboxylate transporters, presenilins, and caveolin-1 [8]. Galectin-3 is a carbohydrate-binding protein whose interaction with CD147 has been shown to stimulate MMP9 expression, thereby destabilizing cell–cell contacts and supporting epithelial motility [15]. In this respect, the abundance of mature N-glycans on CD147 is fundamental to facilitate the interaction with the carbohydrate recognition domain of galectin-3. Our observations suggest that α1,2-mannosidase I participates in this process by allowing the processing of N-glycan precursors in the Golgi that leads to mature forms of CD147. Among terminal structures, poly-N-acetyllactosamine extensions on tri- and tetra-antennary N-glycans are thought to significantly contribute to foster efficient galectin-3 binding [38]. The accumulation of poly-N-acetyllactosamine chains on CD147 has been ascribed to β3GnT8, a glycosyltransferase responsible for transferring N-acetylglucosamine to tetra-antennary N-glycans [39,40]. However, analysis of our PCR array data revealed no obvious upregulation of β3GnT8 during corneal epithelial cell migration, suggesting that the clustering of CD147 by galectin-3 might not require extensive N-acetyllactosamine modification in cornea. In summary, our data indicate that Golgi α1,2-mannosidase I modifies CD147 to stimulate its clustering and biological activities and provide new insights into the importance of mannose-trimming reactions in epithelial wound repair.

## Materials and methods

### Cell culture

Telomerase-immortalized human corneal epithelial cells in tissue culture plates or on glass coverslips were grown in keratinocyte serum-free medium (KSFM; Life Technologies) supplemented with bovine pituitary extract, 0.2 ng/ml epithelium growth factor (EGF) and 0.4 mM CaCl_2_ at 37°C in 5% CO_2_. Once confluent, cells were switched to Dulbecco’s modified Eagle’s medium/F-12 supplemented with 10% newborn calf serum (Thermo Scientific) and 10 ng/mL EGF for 7 d to promote cell stratification and differentiation as described [41]. Where indicated, monolayer or stratified cell cultures were incubated with 8 mM deoxymannojirimycin (DMJ, Tocris Bioscience) or 10 μg/ml tunicamycin (Sigma-Aldrich) for an additional 48 h.

### Generation of wounds

Wounds were generated in stratified cell cultures using a punch injury model as described [18]. For re-epithelialization studies, circular injuries were made in areas equidistant to the center of six-well tissue culture plates using a 3D-printed 8-hole punch template and a 1.0 mm Miltex dermal punch (Integra Miltex). A 33-hole punch template was used when collecting protein cell extracts or total RNA from single wells. Wounds were produced by applying uniform pressure (300–450 g/mm^2^) over the culture for approximately 1 sec while rotating the metal cutting edge of the punch at a 90-degree angle. Cells within the wound were subsequently scraped for approximately 5 sec by rotation of a 10 μl disposable tip connected to a 100-rpm cordless power precision screwdriver. The medium was partially removed before inserting the template into the culture well. Cells at the leading edge were collected by applying a 2.0 mm punch concentric to the wound, followed by mechanical scraping under the microscope using a scalpel blade and aspiration of the material with a sterile pipette. Wounds on glass coverslips (22 × 22 mm) were created using the punch template after placing them in six-well tissue culture plates.

Progress of wound closure was determined by phase-contrast microscopy using an inverted microscope (Nikon Eclipse TS100, Nikon Instruments Inc.) with a 10x objective lens. Morphometric analyses were performed on phase-contrast images of cell cultures immediately after wounding. The perimeter of the wound was manually selected using the freehand tool and the corresponding area quantified with the ‘Measure’ function of ImageJ software (National Institutes of Health; http://rsbweb.nih.gov/ij/). Per each wound, the ratio of re-epithelialization was normalized to the wounded area at time 0 h.

### qPCR and PCR array

Total RNA was isolated using the RNeasy Plus Micro kit (Qiagen) following the manufacturer’s instructions. Five μg (for PCR array) or one μg (for qPCR) total RNA was used for cDNA synthesis (iScript™ cDNA Synthesis; Bio-Rad, Hercules, CA). The qPCR analysis was performed using the SsoAdvanced Universal SYBR Green Supermix (Bio-Rad). Detection of *MMP9, MAN1C1, and CD147* gene expression was performed using PrimePCR™ primers (Unique Assay ID: qHsaCID0011597, qHsaCED0043749, qHsaCID0014293; Bio-Rad). Gene expression was measured in a Mastercycler ep realplex thermal cycler (Eppendorf). The following parameters were used: 2 min at 95°C, followed by 40 cycles of 5 sec at 95°C and 30 sec at 60°C. Expression values were corrected for the housekeeping gene *GAPDH* (Unique Assay ID: qHsaCED0038674; Bio-Rad). The analysis of 84 genes encoding for glycosylation enzymes was carried out using a human glycosylation PCR array (RT^2^ Profiler^TM^ PCR array, SABiosciences Corp.) according to the manufacturer’s instructions. Expression values were corrected for the housekeeping genes *GAPDH* and *RPLP0*. The comparative C_T_ method was used for relative quantitation of the number of transcripts.

### *MAN1C1* knockdown

*MAN1C1* was silenced using a Silencer Pre-designed siRNA (ID133094; Thermo Fisher Scientific). Silencer Negative Control siRNA having no significant similarity to any known gene sequences from mouse, rat, or human was used as a negative control. Preconfluent cultures of human corneal epithelial cells were transfected by a 6-h incubation with 500 nM siRNA in Lipofectamine 2000 (1 μl/100 mm^2^, Life Technologies) dissolved in Opti-MEM reduced-serum medium (Life Technologies) as described [15]. After transfection, the cells were incubated in KSFM for an additional 3 d before treatment with recombinant human galectin-3 (100 µg/ml) or bovine serum albumin control (100 µg/ml) for 24 h.

### Production of recombinant human galectin-3

Recombinant human galectin-3 was produced as previously described [42]. Heterologous protein expression was induced in Rosetta *E. coli* by the addition of 0.3 mM of IPTG. The proteins were purified from lysates by affinity chromatography using lactosyl sepharose. To eliminate contaminating bacterial endotoxins, the proteins were further purified by polymyxin B affinity chromatography (Sigma-Aldrich). The absence of lipopolysaccharide was confirmed using the ToxinSensor^TM^ Chromogenic LAL Endotoxin Assay Kit (GenScript, Piscataway, NJ, USA). Protein solutions were concentrated by centrifugal filtration (VWR, Radnor, PA, USA), dialyzed against PBS buffer containing 10% glycerol, and stored at −20°C.

### Immunofluorescence

Cell cultures were fixed with 4% formaldehyde for 10 min, washed with PBS, and permeabilized with 1% Triton X-100 for 10 minutes. Following hydration in PBS for 5 min, cultures were blocked in PBS containing 1% bovine serum albumin for 10 min, followed by incubation with a primary anti-CD147 antibody (1:500; Biolegend, or 1:100; Clone A-12, Santa Cruz Biotechnology) overnight at 4°C. Incubation with the primary antibody was routinely omitted in control experiments. After washing with PBS, the corresponding secondary antibody (Alexa Fluor 488-conjugated goat anti-mouse IgG; 1:500 or 1:1,000) was applied for 1 h at room temperature. Coverslips were then washed, mounted in VectaShield mounting medium containing DAPI (Vector Laboratories), and imaged on a Zeiss Axio Observer Z1 inverted fluorescent microscope (Carl Zeiss Microimaging GmbH). Analysis of CD147 fluorescence intensity was performed using ImageJ software (National Institutes of Health, Bethesda, MD).

### In situ zymography

The in situ gelatinolytic activity was measured using the EnzChek Gelatinase Assay kit (Molecular Probes) following the manufacturer’s instructions. Briefly, stratified cell cultures on glass coverslips were incubated with DQ-gelatin-fluorescein isothiocyanate for 24 h at 37°C, washed with PBS, and mounted in VectaShield mounting medium containing DAPI. Sections were then examined on a Zeiss Axio Observer Z1 inverted fluorescent microscope and photographed. As a negative control, cultures were incubated with reaction buffer supplemented with a general metalloproteinase inhibitor (1,10-phenanthroline).

### Immunoblotting

Protein from cell cultures was extracted using a buffer containing 150 mM NaCl, 50 mM Tris-HCl, pH 8.0, 1% NP-40, 0.5% deoxycholate, and 0.1% SDS supplemented with Complete™ EDTA-free Protease Inhibitor Cocktail (Roche Diagnostics). After homogenization with a pellet pestle, the protein cell extracts were centrifuged at 12,000 g for 45 min at 4°C, and the protein concentration of the supernatant determined using the Pierce BCA™ Protein Assay Kit (Thermo Fisher Scientific). Proteins in cell lysates (20–40 μg) were resolved in 10% SDS-PAGE and electroblotted onto nitrocellulose membranes (Bio-Rad). Membranes were then incubated with primary antibodies against CD147 (clone A-12, 1:3,000, Santa Cruz Biotechnology) or β-actin (1:3,000, Cell Signaling Technologies) in TTBS supplemented with 5% nonfat dry milk overnight at 4°C, followed by the secondary antibody coupled to horseradish peroxidase (1:2,000, Santa Cruz Biotechnology). Peroxidase activity was visualized using SuperSignal West Pico Chemiluminescent Substrate (Thermo Fisher Scientific) and imaged using the G:BOX Chemi XRQ (Syngene).

### Statistical analysis

All statistical analyses were performed using Prism 7 (Graphpad Software) for Mac OSX.
