# Supplementary material for: Golgi α1,2-mannosidase I induces clustering and compartmentalization of CD147 during epithelial cell migration
Source: Cell Adh Migr. 2020 May 18;14(1):96–105. doi: 10.1080/19336918.2020.1764170 (PMC7250185; doi:10.1080/19336918.2020.1764170)
Supplement: Supplemental Material [file kcam-14-01-1764170-s001.docx]

| **Supplemental Table S1.** Relative expression of genes encoding glycosylation enzymes in stratified human corneal epithelial cells. Cell cultures were wounded using a 33-hole punch template and analyzed 48 h later. Unwounded cell cultures were used as control. Values in bold indicate that gene expression was higher or lower than 1.5-fold compared to control. High ΔC_T_ values reflect low amplification efficiency. | | | | |
| --- | --- | --- | --- | --- |
| **Gene Symbol** | **RefSeq*** | | **Magnitude of expression in Control (ΔC_T_)** | **Fold change**  **(Wound/Control)** |
| *N-Acetylgalactosaminyltransferases* | | |  |  |
| *GALNT1* | NM_020474 | | 5.87 | 0.93 |
| *GALNT2* | NM_004481 | | 7.22 | **0.63** |
| *GALNT3* | NM_004482 | | 4.89 | 1.21 |
| *GALNT4* | NM_003774 | | 10.64 | 0.86 |
| *GALNTL5* | NM_145292 | | 19.78 | 1.04 |
| *GALNT6* | NM_007210 | | 9.61 | 0.97 |
| *GALNTL6* | NM_001034845 | | 15.90 | 1.49 |
| *GALNT7* | NM_017423 | | 7.20 | 1.14 |
| *GALNT8* | NM_017417 | | 18.51 | 0.81 |
| *GALNT9* | NM_021808 | | 19.78 | 1.04 |
| *GALNT10* | NM_198321 | | 9.99 | 0.82 |
| *GALNT11* | NM_022087 | | 8.58 | 1.16 |
| *GALNT12* | NM_024642 | | 11.24 | 1.26 |
| *GALNT13* | NM_052917 | | 9.57 | 1.16 |
| *GALNT14* | NM_024572 | | 9.16 | 1.01 |
| *GALNT16* | NM_020692 | | 17.13 | 0.75 |
| *N-Acetylglucosaminyltransferases* | | |  |  |
| *A4GNT* | NM_016161 | | 19.07 | 1.21 |
| *B3GNT2* | NM_006577 | | 6.99 | 1.06 |
| *B3GNT3* | NM_014256 | | 11.51 | 0.82 |
| *B3GNT4* | NM_030765 | | 13.42 | 0.76 |
| *B3GNT8* | NM_198540 | | 14.06 | 1.17 |
| *GCNT1* | NM_001490 | | 8.32 | 1.22 |
| *GCNT3* | NM_004751 | | 11.57 | **1.82** |
| *GCNT4* | NM_016591 | | 9.90 | 1.07 |
| *MGAT1* | NM_002406 | | 7.60 | **0.62** |
| *MGAT2* | NM_002408 | | 12.32 | **0.62** |
| *MGAT3* | NM_002409 | | 19.42 | 1.16 |
| *MGAT4A* | NM_012214 | | 8.70 | **1.56** |
| *MGAT4B* | NM_014275 | | 6.94 | 1.20 |
| *MGAT4C* | NM_013244 | | 17.25 | **1.89** |
| *MGAT5* | NM_002410 | | 8.84 | 0.89 |
| *MGAT5B* | NM_144677 | | 17.16 | 1.07 |
| *OGT* | NM_181673 | | 4.88 | 1.22 |
| *POMGNT1* | NM_017739 | | 8.37 | 0.85 |
| *Galactosyltransferases* | | |  |  |
| *B4GALT1* | NM_001497 | | 6.14 | 0.82 |
| *B4GALT2* | NM_003780 | | 7.21 | 0.80 |
| *B4GALT3* | NM_003779 | | 7.34 | 1.04 |
| *B4GALT5* | NM_004776 | | 5.84 | 1.09 |
| *C1GALT1* | NM_020156 | | 6.90 | 1.39 |
| *Glucosyltransferases* | | |  |  |
| *UGGT1* | NM_020120 | | 7.39 | 0.96 |
| *UGGT2* | NM_020121 | | 7.21 | 1.04 |
| *Mannosidases* |  | |  |  |
| *EDEM1* | NM_014674 | | 7.13 | 0.93 |
| *EDEM2* | NM_018217 | | 7.23 | 1.00 |
| *EDEM3* | NM_025191 | | 8.86 | 0.88 |
| *MAN1A1* | NM_005907 | | 7.00 | 1.11 |
| *MAN1A2* | NM_006699 | | 7.05 | 0.95 |
| *MAN1B1* | NM_016219 | | 8.70 | 0.85 |
| *MAN1C1* | NM_020379 | | 18.03 | **2.24** |
| *MAN2A1* | NM_002372 | | 5.96 | 0.94 |
| *MAN2A2* | NM_006122 | | 11.93 | 0.92 |
| *MAN2B1* | NM_000528 | | 7.32 | 1.36 |
| *MANBA* | NM_005908 | | 6.15 | 1.19 |
| *Mannosyltransferases* | | |  |  |
| *POMT1* | NM_007171 | | 9.39 | 0.97 |
| *POMT2* | NM_013382 | | 8.46 | 0.83 |
| *Galactosidases, glucosidases and hexosaminidases* | | | |  |
| *GANAB* | NM_198334 | 6.48 | | 0.70 |
| *GLB1* | NM_000404 | 7.08 | | 1.08 |
| *HEXA* | NM_000520 | 6.92 | | 1.38 |
| *HEXB* | NM_000521 | 4.34 | | 1.08 |
| *MOGS* | NM_006302 | 9.73 | | **0.60** |
| *PRKCSH* | NM_002743 | 6.21 | | 0.84 |
| *Fucosidases and fucosyltransferases* | | | |  |
| *FUCA1* | NM_000147 | 8.28 | | 1.19 |
| *FUCA2* | NM_032020 | 6.64 | | 1.08 |
| *FUT8* | NM_178157 | 8.27 | | 1.40 |
| *FUT11* | NM_173540 | 6.59 | | **0.52** |
| *POFUT1* | NM_172236 | 10.48 | | 0.77 |
| *POFUT2* | NM_133635 | 11.18 | | 0.81 |
| *Sialidases* | | | |  |
| *NEU1* | NM_000434 | 6.07 | | 0.97 |
| *NEU2* | NM_005383 | 17.97 | | 1.19 |
| *NEU3* | NM_006656 | 11.30 | | 0.76 |
| *NEU4* | NM_080741 | 19.78 | | 1.04 |
| *Sialyltransferases* | | | |  |
| *ST3GAL1* | NM_173344 | 9.91 | | 0.82 |
| *ST3GAL2* | NM_006927 | 11.88 | | 0.81 |
| *ST6GAL1* | NM_003032 | 10.47 | | 1.32 |
| *ST6GALNAC1* | NM_018414 | 12.64 | | 0.91 |
| *ST8SIA2* | NM_006011 | 19.68 | | 1.04 |
| *ST8SIA3* | NM_015879 | 19.78 | | **1.62** |
| *ST8SIA4* | NM_175052 | 13.06 | | **1.57** |
| *ST8SIA6* | NM_001004470 | 15.70 | | 1.25 |
| *Mannose-6-phosphate synthesis and catabolism* | | | |  |
| *GNPTAB* | NM_024312 | 6.88 | | 1.24 |
| *GNPTG* | NM_032520 | 5.96 | | 1.19 |
| *NAGPA* | NM_016256 | 12.36 | | 0.93 |
| *Other glycosylation genes* | |  | |  |
| *AGA* | NM_000027 | 6.71 | | 1.01 |
| *C1GALT1C1* | NM_152692 | 6.46 | | 1.04 |

*Reference sequence database at NCBI (https://www.ncbi.nlm.nih.gov/refseq/)
